# Supplementary figures and images for: Comparative Analysis of the Ecological Succession of Microbial Communities on Two Artificial Reef Materials
Source: Microorganisms. 2021 Jan 6;9(1):120. doi: 10.3390/microorganisms9010120 (PMC7825563; doi:10.3390/microorganisms9010120)

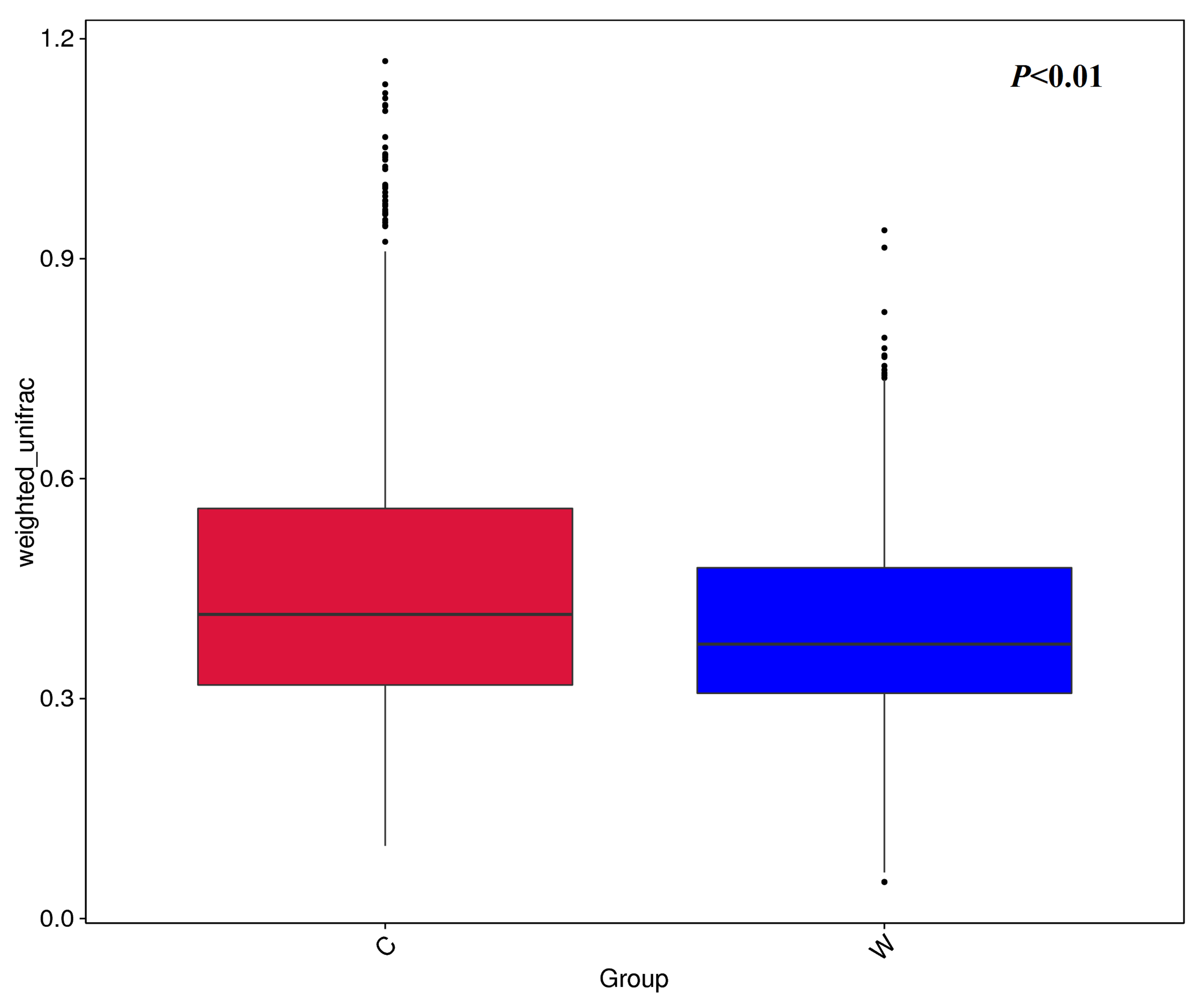

Supplement: Supplementary file 1 [file microorganisms-09-00120-s001.zip › microorganisms-1035447-supplementary/Fig S1.jpg]
