# Supplementary material for: Comparative Analysis of the Ecological Succession of Microbial Communities on Two Artificial Reef Materials
Source: Microorganisms. 2021 Jan 6;9(1):120. doi: 10.3390/microorganisms9010120 (PMC7825563; doi:10.3390/microorganisms9010120)
Supplement: Supplementary file 1 [file microorganisms-09-00120-s001.zip › microorganisms-1035447-supplementary/Table S1.doc]

Table 1S Alpha diversity indices of microbial community

| Group | OTU Number | Observed Species | Shannon | chao1 | Goods Coverage |
| --- | --- | --- | --- | --- | --- |
| C1 | 2216.33 | 1575 | 7.43 | 2195.20 | 0.97 |
| C2 | 2242.33 | 1587 | 7.74 | 2052.12 | 0.98 |
| C3 | 2827.50 | 2101 | 8.80 | 2937.93 | 0.96 |
| C4 | 2051.33 | 1630 | 7.53 | 2133.19 | 0.98 |
| C5 | 2568.33 | 1932 | 8.67 | 2662.38 | 0.97 |
| C6 | 2634.00 | 2008 | 8.77 | 2783.98 | 0.97 |
| C7 | 3592.33 | 2655 | 9.40 | 3768.27 | 0.96 |
| C8 | 2618.00 | 1938 | 8.75 | 2456.04 | 0.97 |
| C9 | 2385.50 | 1869 | 8.44 | 2435.36 | 0.97 |
| C10 | 2244.33 | 1843 | 8.87 | 2175.08 | 0.98 |
| C11 | 2705.67 | 1987 | 8.57 | 2575.83 | 0.97 |
| W1 | 1647.33 | 1213 | 7.25 | 1644.19 | 0.98 |
| W2 | 1904.33 | 1374 | 7.44 | 1831.52 | 0.98 |
| W3 | 2566.33 | 1796 | 8.15 | 2519.86 | 0.97 |
| W4 | 1526.67 | 1253 | 6.61 | 2432.79 | 0.98 |
| W5 | 2097.67 | 1447 | 7.59 | 2009.23 | 0.98 |
| W6 | 2238.67 | 1602 | 7.73 | 2050.63 | 0.98 |
| W7 | 2815.00 | 1988 | 8.48 | 2847.81 | 0.97 |
| W8 | 2179.33 | 1685 | 8.13 | 2314.80 | 0.97 |
| W9 | 2328.33 | 1621 | 7.82 | 2236.88 | 0.97 |
| W10 | 1961.67 | 1440 | 7.65 | 1900.52 | 0.98 |
| W11 | 1607.67 | 1307 | 7.19 | 1733.06 | 0.98 |
